# Miniaturized Rotary Actuators Using Shape Memory Alloy for Insect-Type MEMS Microrobot

**DOI:** 10.3390/mi7040058

**Published:** 2016-03-31

**Authors:** Ken Saito, Kei Iwata, Yuki Ishihara, Kazuki Sugita, Minami Takato, Fumio Uchikoba

**Affiliations:** 1 Department of Precision Machinery Engineering, College of Science and Technology, Nihon University, 7-24-1 Narashinodai, Funabashi-shi, Chiba 274-8501, Japan; takato@eme.cst.nihon-u.ac.jp (M.T.); uchikoba@eme.cst.nihon-u.ac.jp (F.U.); 2 Precision Machinery Engineering, Graduate School of Science and Technology, Nihon University, 1-8-14 Kanda Surugadai, Chiyoda-ku, Tokyo 101-8308, Japan; cske14010@g.nihon-u.ac.jp (K.I.); csyu14004@g.nihon-u.ac.jp (Y.I.); cska15018@g.nihon-u.ac.jp (K.S.)

**Keywords:** microrobot, rotary actuator, shape memory alloy, artificial neural networks, MEMS, heat conduction

## Abstract

Although several types of locomotive microrobots have been developed, most of them have difficulty locomoting on uneven surfaces. Thus, we have been focused on microrobots that can locomote using step patterns. We are studying insect-type microrobot systems. The locomotion of the microrobot is generated by rotational movements of the shape memory alloy-type rotary actuator. In addition, we have constructed artificial neural networks by using analog integrated circuit (IC) technology. The artificial neural networks can output the driving waveform without using software programs. The shape memory alloy-type rotary actuator and the artificial neural networks are constructed with silicon wafers; they can be integrated by using micro-electromechanical system (MEMS) technology. As a result, the MEMS microrobot system can locomote using step patterns. The insect-type MEMS microrobot system is 0.079 g in weight and less than 5.0 mm in size, and its locomotion speed is 2 mm/min. The locomotion speed is slow because the heat of the shape memory alloy conducts to the mechanical parts of the MEMS microrobot. In this paper, we discuss a new rotary actuator compared with the previous model and show the continuous rotation of the proposed rotary actuator.

## 1. Introduction

Many kinds of microrobot systems have been studied by several papers [1,2,3,4,5,6,7,8]. Especially, microrobot systems which are less than 5 cm in size have attracted attention [4,5,6,7,8]. The microrobot will be useful for several applications such as manipulation or assembly of small-sized components. However, higher functionalization and further miniaturization are desired in the microrobot. In contrast, to realize their autonomous operation, insects have excellent structures and use advanced neural networks controlled by small-sized systems. Therefore, many studies of microrobots were biologically inspired to realize the excellent functions of insects. The miniaturization of the microrobot system has been done using mechanical machining. There are some difficulties regarding actuators, frame parts, motion controllers, sensors, and power sources in order to achieve further miniaturization [9]. Instead of mechanical machining, microfabrication technology based on integrated circuit (IC) technology has been used to make the miniaturized actuators [10,11]. The type of the miniaturized actuators using microfabrication technology is categorized into two groups: one using the property of the material itself and the other using field forces [12,13,14,15,16]. However, most of the miniaturized actuators of the microrobot have weakness in locomoting on uneven surfaces. Therefore, a microrobot which can locomote using a step pattern has been focused on.

Some of the latest studies of artificial neural networks have attracted attention for application in robot control [17,18,19]. Several studies have reported both on hardware models and on software models. However, the large-scale artificial neural networks constructed by software models are difficult to implement to the microcontroller. In contrast, the large-scale artificial neural networks constructed by hardware models are advantageous because the IC technology could reduce the circuit scale. In addition, IC can be combined with mechanical parts of the microrobot using micro-electromechanical system (MEMS) technology. Therefore, MEMS technology can reduce the size of the robot at the millimeter scale.

We are studying microrobot systems which can locomote using step patterns. In addition, we are constructing artificial neural networks which can generate the locomotion of the robot. A microrobot of 4.0 mm in width, 4.0 mm in length, and 3.5 mm in height, and artificial neural networks constructed by a discrete circuit board were reported in a previous work [20]. The microrobot had been downsized to 4.0 mm in width, 2.7 mm in length and 2.5 mm in height in a later work [21]. In addition, artificial neural networks were constructed by IC for the purpose of minimizing the circuit size. However, a peripheral circuit was needed to drive the shape memory alloy-type rotary actuator in the previous IC chip of artificial neural networks. The peripheral circuit consisted of the transistors to amplify the electric current and the operational amplifier for the buffer. The weight of the MEMS microrobot system was 0.33 g (IC with peripheral circuit: 0.31 g; MEMS microrobot: 0.02 g). The MEMS microrobot system could not locomote because the IC with the peripheral circuit was too heavy. Therefore, a reduction in size and weight are needed for the IC with a peripheral circuit [22,23]. In addition, the locomotion speed becomes slow after 30 s.

In this paper, we discuss a new rotary actuator compared with a previous model. Firstly, a micro-mechanical system of the MEMS microrobot is shown briefly. (The basic concept of the MEMS microrobot was shown in [21]. The difference is only the rotary actuator.) Secondly, a micro-electro system of the MEMS microrobot using artificial neural networks is discussed. (The basic concept of the artificial neural networks was shown in [22,23]. The difference is only that the artificial neural networks are mountable to the robot.) Finally, we show the locomotion of the insect-type MEMS microrobot system. Additionally, we analyze the heat conduction of the shape memory alloy-type rotary actuator.

## 2. Materials and Methods

### 2.1. Micro-Mechanical System

We used microfabrication technology to construct the micro-mechanical system of the MEMS microrobot. The rotors of the shape memory alloy-type rotary actuators, body frame, link mechanisms and six legs are made from silicon wafers. The shapes of the micro-mechanical systems are machined by inductively coupled plasma (ICP) dry etching with photolithography technology [24].

#### 2.1.1. Mechanical Components

**Figure 1:**
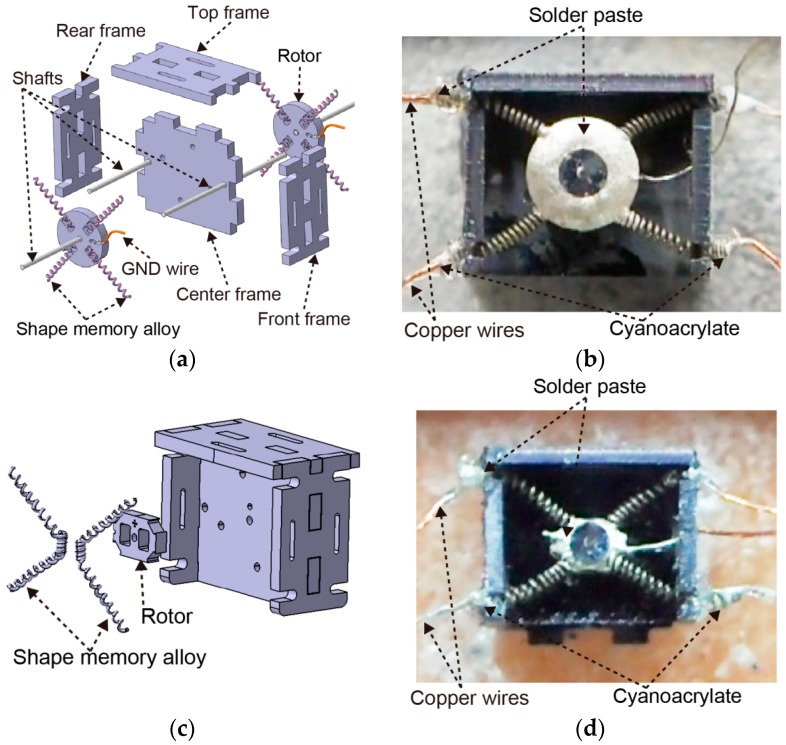
Shape memory alloy-type rotary actuator: (**a**) Exploded view (old type); (**b**) Picture after being assembled (old type); (**c**) Exploded view (new type); (**d**) Picture after being assembled (new type).

Figure 1 shows the shape memory alloy-type rotary actuator of the MEMS microrobot. Figure 1a shows the exploded view of the old type of rotary actuator and Figure 1b shows the picture after being assembled. Figure 1c shows the exploded view of the new type of rotary actuator and Figure 1d shows the picture after being assembled. The differences of the two types of the rotary actuators are the shape of the rotor and the number of the shape memory alloys. The old type of rotary actuator uses four pieces of shape memory alloy while the new type of rotary actuator uses two pieces of shape memory alloy. Both types of the rotary actuators are assembled with exactly the same frame parts. The frame parts consist of a center frame, a front frame, a rear frame and a top frame. The rotary actuators consist of shape memory alloy, shaft, ground (GND) wire, and a rotor. The rotary actuator uses the shape memory alloy to generate the rotational movement which is necessary for the locomotion of the microrobot. The shape memory alloy can change in length according to the temperature (over 70 °C: shrink; under 70 °C: extend). Therefore, our constructed actuator could not rotate if the temperature of the shape memory alloy could not cool to less than 70 °C. In this study, the length of the shape memory alloy was changed by supplying or stopping the current flow. One side of the shape memory alloy is connected to the rotor and the other side is connected to the copper wires by using solder paste (Figure 1b,d). The GND wire is fixed to the rotor directly. The rotor of the rotary-type actuator is connected by the shape memory alloy to the frame components. The shape memory alloy used in this study is BioMetal^®^ Helix BMX50 (Toki Corporation, Tokyo, Japan). The basic characteristics of the shape memory alloy were as follows. The standard coil diameter of the shape memory alloy was 0.2 mm and the wire diameter was 0.05 mm. The practical maximum force produced 3 to 5 gram-force (gf) where kinetic displacement was 50%. The standard drive current was 50 to 100 mA where standard electric resistance was 3600 ohm/m.

**Figure 2:**
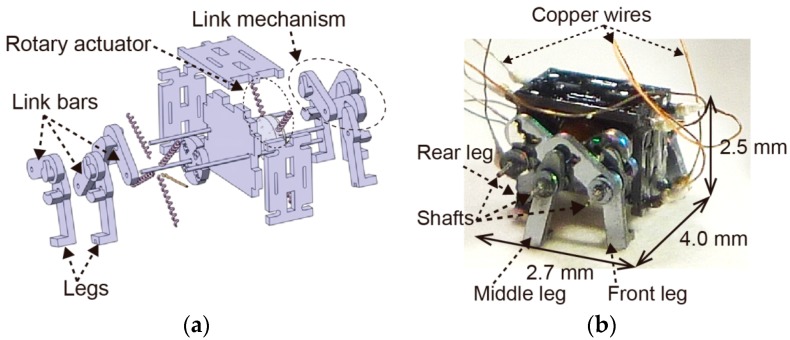
Micro-mechanical system of the fabricated MEMS microrobot: (**a**) Exploded view; (**b**) Picture after being assembled.

Figure 2 shows the micro-mechanical system of the fabricated MEMS microrobot. Figure 2a shows the exploded view and Figure 2b shows the picture after being assembled. The link mechanism consists of three legs, shafts and link bars. The middle legs are fixed to the rotor using the shafts; therefore, the middle legs and the rotors have the same rotational phases. By contrast, the front legs and the rear legs are fixed to the middle legs by link bars using shafts; therefore, the front legs and the rear legs can generate a ±90° phase shift compared with the middle legs. The MEMS microrobot measures 4.0 mm in width, 2.7 mm in length, 2.5 mm in height. The copper wires of Figure 2b show the eight signal wires and two GND wires; the MEMS microrobot can locomote when the signal wires are connected to the artificial neural networks.

#### 2.1.2. Locomotion Mechanism Using Shape Memory Alloy-Type Rotary Actuator

We replicate the locomotion mechanisms of an ant using link mechanisms and shape memory alloy-type rotary actuators.

**Figure 3:**
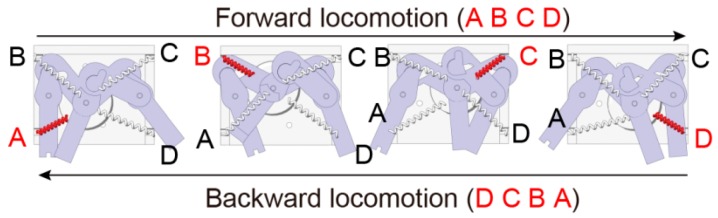
Schematic diagram of the locomotion mechanism.

Figure 3 shows the schematic diagram of the locomotion mechanism. The MEMS microrobot can locomote from the rotation of the rotary actuator. In the case of heating the shape memory alloy wires from A to D, the microrobot performs forward locomotion. In contrast, when heating the shape memory alloy from D to A, the microrobot performs backward locomotion. The locomotion pattern of the left-side legs and the right-side legs has a 180° phase shift.

### 2.2. Micro-Electro System

We constructed the artificial neural networks using the pulse-type hardware neuron model as the basic components. The pulse-type hardware neuron model is a class II neuron model. The class II neuron model has the same features as biological neurons such as spatio-temporal summation characteristics, refractory period and threshold. Therefore, the pulse-type hardware neuron model could generate continuous pulse waves as biological neurons do.

#### 2.2.1. Electrical Components

**Figure 4:**
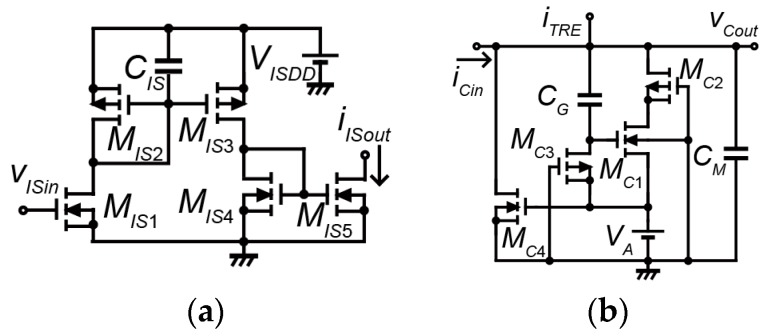
Circuit diagram of the pulse-type hardware neuron model: (**a**) Inhibitory synaptic model; (**b**) Cell body model.

Figure 4 shows the circuit diagram of the pulse-type hardware neuron model. The pulse-type hardware neuron model consists of a synaptic model and a cell body model. Figure 4a,b show the circuit diagrams of the inhibitory synaptic model and the cell body model, respectively. The synchronization phenomena of the cell body model could change from the coupling of the synaptic model. Coupling by the excitatory synaptic model and coupling by the inhibitory synaptic model causes in-phase synchronization and anti-phase synchronization, respectively (please see [25] for more detail). The circuit parameters of the synaptic model are as follows: *C_IS_* = 1 pF, *M_IS_*_1–5_: W/L = 1 (where W is an effective channel width and L is an effective channel length of the metal-oxide semiconductor field-effect transistor (MOSFET)). The voltage source *V_ISDD_* was set to 5 V. The circuit parameters of the cell body model are as follows: *C_G_* = 39 µF, *C_M_* = 270 nF, *M_C_*_1_, *M_C_*_2_: W/L = 10, *M_C_*_3_: W/L = 0.1, *M_C_*_4_: W/L = 0.3. The voltage source *V_A_* was set to 3.3 V. The input voltage *v_ISin_* of the synaptic model is the output voltage *v_Cout_* of the other cell body model. The input current *i_Cin_* of the cell body model is the output current *i_ISout_* of the synaptic model.

#### 2.2.2. Artificial Neural Networks

**Figure 5:**
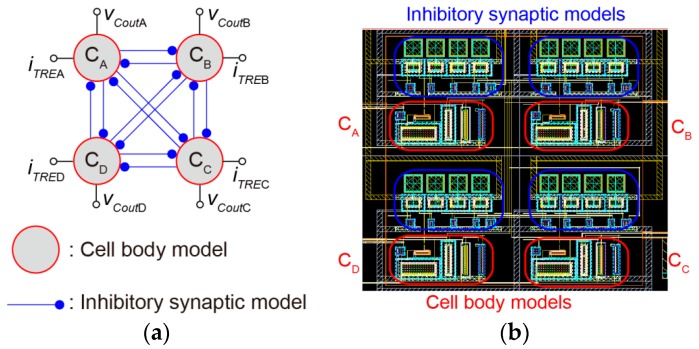
Artificial neural networks: (**a**) Connection diagram; (**b**) Layout pattern.

We designed the artificial neural networks using inhibitory mutual coupling which could generate the anti-phase synchronization. The constructed artificial neural networks are shown in Figure 5. Figure 5a shows the connection diagram and Figure 5b shows the layout pattern of the artificial neural networks, respectively. The four cell body models are mutually coupled by 12 inhibitory synaptic models. In Figure 5a, *v_Cout_*_A_, *v_Cout_*_B_, *v_Cout_*_C_ and *v_Cout_*_D_ indicate the output ports of the artificial neural networks. In addition, *i_TRE_*_A_, *i_TRE_*_B_, *i_TRE_*_C_ and *i_TRE_*_D_ indicate the trigger pulse input ports of the artificial neural networks. The artificial neural networks can change the sequence of driving pulses according to the input timing of a single external trigger pulse. The design rule of the IC is four metal, two poly complementary metal oxide semiconductor (CMOS) 0.35 μm.

#### 2.2.3. Current Mirror Circuit

The cell body model outputs the voltage waveform. However, the shape memory alloy-type actuators need an electrical current to generate the rotational movements. Therefore, we convert the voltage waveform to the current waveform.

**Figure 6:**
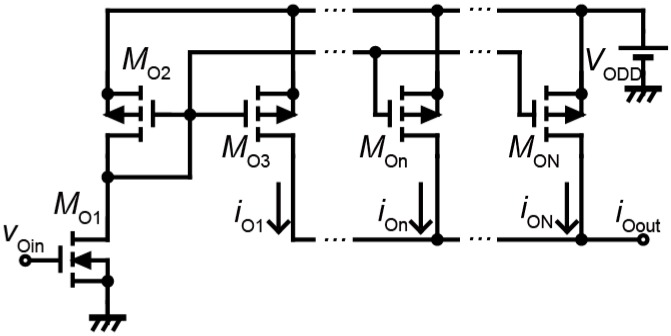
Circuit diagram of the current mirror circuit.

Figure 6 shows the circuit diagram of the current mirror circuit. The current mirror circuit generates the output current waveform *i*_O*out*_ according to the voltage waveform *v*_C*out*_ (*v*_O*in*_). The circuit parameters of the current mirror circuit are as follows: *M*_1_: W/L = 40, *M*_2_: W/L = 1, *M*_O*n*_: W/L = 66.7. In this paper, we set it as *N* = 11 (3 ≤ *n* ≤ *N*) because the wire bonding between the bare chip IC and the robot can only output 50 mA.

#### 2.2.4. Bare Chip IC of Artificial Neural Networks with Current Mirror Circuit

We constructed the bare chip IC of artificial neural networks with a current mirror circuit. The bare chip IC is fixed to the cavity of the flame retardant 4 (FR4) substrate. Each pad of the bare chip IC is connected to the pad pattern of the FR4 substrate. We use the ultrasonic wire bonding to fix the aluminum wire.

**Figure 7:**
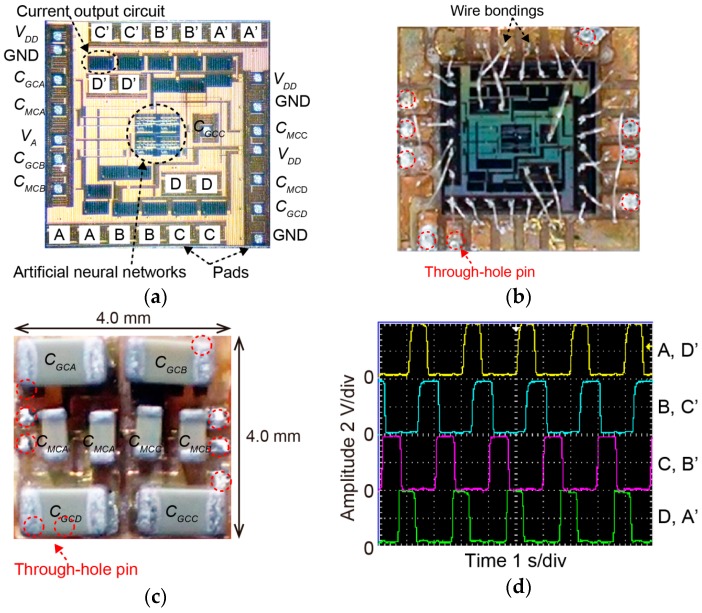
Bare chip IC of artificial neural networks with current output circuit: (**a**) Picture of the bare chip IC; (**b**) Picture of the bare chip IC with FR4 substrate (top side); (**c**) Picture of the bare chip IC with FR4 substrate (bottom side); (**d**) Output waveform of the bare chip IC (measured value).

Figure 7 shows the bare chip IC of artificial neural networks with the current output circuit. Figure 7a shows the bare chip IC. Figure 7b,c show the top side and bottom side of the bare chip IC with FR4 substrate, respectively. Figure 7d shows the output waveform of the bare chip IC (measured value). In Figure 7a, several pads are arranged to fix the wire bonding. In addition, two pads are placed for each port of the microrobot. This is because the shape memory alloy-type actuator needs a large current to actuate. The capacitors of the cell body model were impossible to implement inside the bare chip IC because of the size. Therefore, the capacitors *C_G_* and *C_M_* are mounted externally of the bare chip IC as shown in Figure 7c. Artificial neural networks are coupled neural network systems which can generate the locomotion rhythms as living organisms. To heat the helical artificial muscle wires, the input of the pulse 0.5 s in width, 2 s in period and 75 mA in amplitude is required. According to Figure 7d, the output waveform is enough to actuate the rotary actuator because the resistance of the shape memory alloy-type rotary actuator is about 50 Ω.

## 3. Results and Discussion

**Figure 8:**
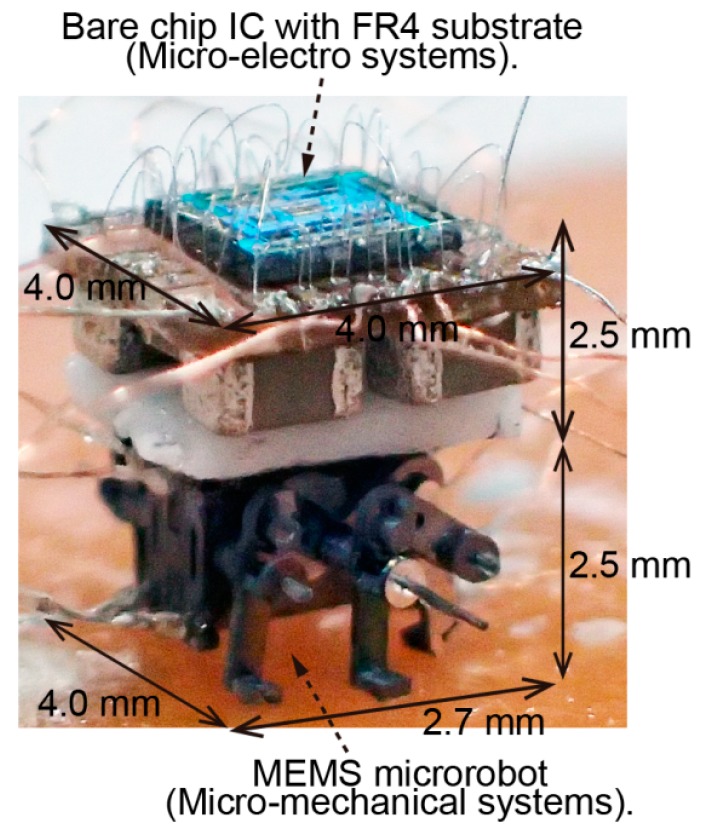
Assembled MEMS microrobot system.

Figure 8 shows the assembled MEMS microrobot system. We mount the bare chip IC with FR4 substrate on top of the microrobot. The whole system is 0.079 g in weight and less than 5.0 mm in size (micro-mechanical system: 0.02 g in weight, 4.0 mm in width, 2.7 mm in length and 2.5 mm in height; micro-electro system: 0.059 g in weight, 4.0 mm in width, 4.0 mm in length and 2.5 mm in height).

### 3.1. Locomotion of the MEMS Microrobot System

**Figure 9:**
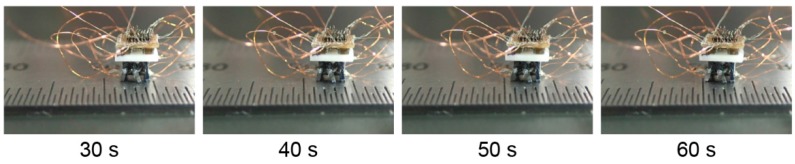
Example of locomotion of the MEMS microrobot system.

Figure 9 shows the example of locomotion of the MEMS microrobot system. Artificial neural networks can generate the driving pulse which is necessary to locomote the MEMS microrobot. The locomotion speed of the MEMS microrobot system is 4 mm/min. The MEMS microrobot system without mounting the bare chip IC with FR4 substrate was 26.4 mm/min [21]. The locomotion of the MEMS microrobot system is too slow compared with not mounting the bare chip IC with FR4 substrate. One of the reasons for the slow locomotion speed is the heat conduction of the shape memory alloy-type rotary actuator.

### 3.2. Heat Conduction of the Shape Memory Alloy-Type Rotary Actuator

**Figure 10:**
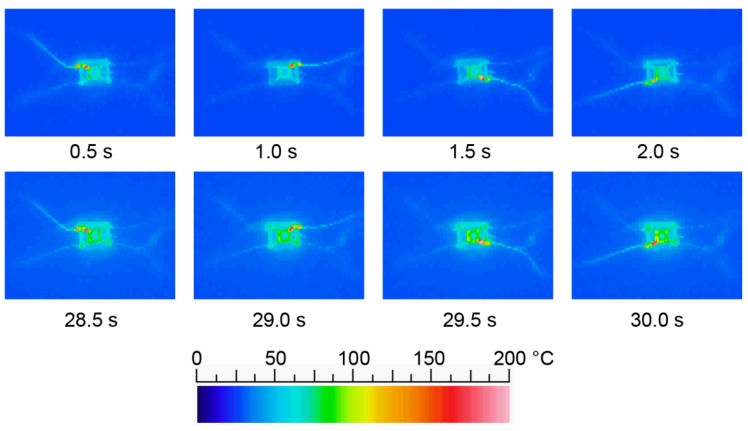
Heat conduction of the shape memory alloy-type rotary actuator.

Figure 10 shows the heat conduction of the shape memory alloy-type rotary actuator. The heat of the shape memory alloy conducts to the mechanical components of the MEMS microrobot. The shape memory alloy shrinks at temperatures over 70 °C and extends at temperatures under 70 °C. The thermal difference needs to be extended. However, the shape memory alloy heats up to over 80 °C after 30 s. The heat conduction interferes with the radiation of the shape memory alloy. Therefore, locomotion becomes slow after 30 s. To solve this problem, we constructed a new type of rotary actuator to reduce the heat conduction. The thermal conductivity of each material is as follows: solder paste, 10.0 W/(m·K); shape memory alloy, 12.1 W/(m·K); Si, 168 W/(m·K). According to the thermal conductivity of each material, the solder paste and shape memory alloy keep the heat.

**Figure 11:**
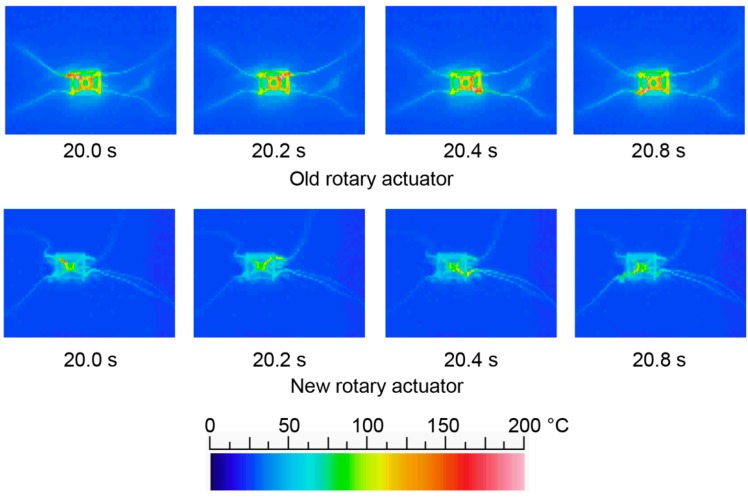
Heat conduction of the new type of rotary actuator compared with the old type.

Figure 11 shows the heat conduction of the new type of rotary actuator compared with the old type. The pulse width is set as 0.2 s and the pulse period is set as 0.8 s (2.5 times shorter than Figure 7d). It is shown that the old type of rotary actuator cannot radiate under 100 °C; the shape memory alloy cannot generate the rotational movement. On the other hand, the new type of rotary actuator can keep the temperature under 70 °C; the shape memory alloy can generate the rotational movement. Therefore, locomotion can keep its speed after 30 s.

**Figure 12:**
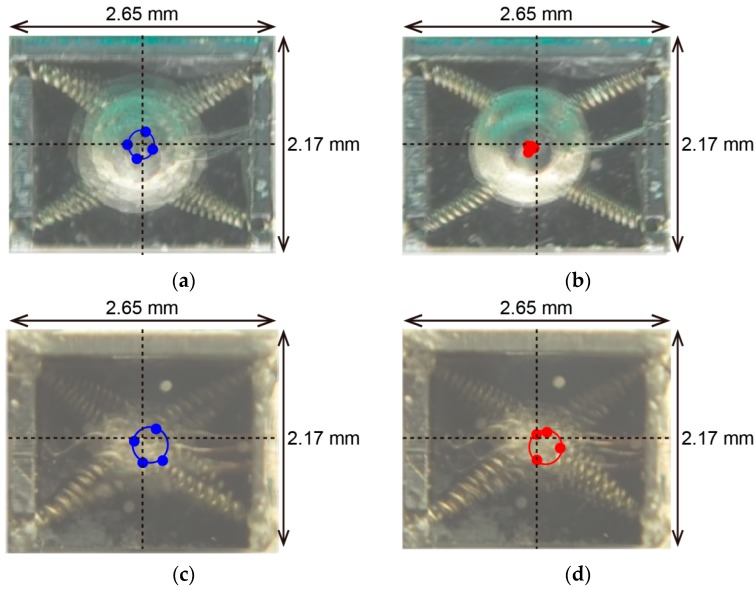
Movements of the rotor of the new type of rotary actuator comparing with the old type: (**a**) Movements of the rotor of the old type of rotary actuator (before 10 s); (**b**) Movements of the rotor of the old type of rotary actuator (after 30 s); (**c**) Movements of the rotor of the new type of rotary actuator (before 10 s); (**d**) Movements of the rotor of the new type of rotary actuator (after 30 s).

Figure 12 shows the movements of the rotor of the new type of rotary actuator compared with the old type. Figure 12a shows the movements of the rotor of the old type of rotary actuator (before 10 s), and Figure 12b shows the movements of the rotor of the old type of rotary actuator (after 30 s). Figure 12c shows the movements of the rotor of the new type of rotary actuator (before 10 s), and Figure 12d shows the movements of the rotor of the new type of rotary actuator (after 30 s). In these figures, solid circles indicate the center of the rotor and solid lines indicate the trajectory of the center of the rotor. It is shown that the new type of rotary actuator can generate continuous rotational movements after 30 s. The displacement of the shape memory alloy depends on its temperature. The displacement *versus* time of the shape memory alloy is about 0.63 mm/s (measurement value of Figure 12, where the pulse amplitude is 4 V, the pulse width is 0.2 s and the pulse period is 0.8 s.). The rotary rate of the rotor depends on the pulse period. For example, it is 30 rpm in the case of a pulse period that is 2 s and it is 75 rpm in the case of a pulse period that is 0.8 s. The maximum force produced by the shape memory alloy is 5 gf. The power consumption of the shape memory alloy-type rotary actuator depends on its temperature. The power consumption is about 80–400 mWh.

## 4. Conclusions

In this paper, we discussed miniaturized rotary actuators for insect-type MEMS microrobots. We studied the new rotary actuator compared with the previous model.

As a result, the MEMS microrobot system can locomote using step patterns. The insect-type MEMS microrobot system is 0.079 g in weight and less than 5.0 mm in size. The locomotion speed is 2 mm/min. However, the locomotion speed is slow because the heat of the shape memory alloy conducts to the mechanical parts of the MEMS microrobot. It was shown that the new type of rotary actuator could generate continuous rotational movements.

Now we are constructing the new MEMS microrobot system using the new type of rotary actuator which can generate continuous locomotion. In the future, the artificial neural networks, power source system and sensory system will directly integrate on the frame parts of the microrobot. In addition, we will consider not using the manual assembly step to construct the microrobot by batch fabrication.

## Supplementary Materials

The following are available online at http://www.mdpi.com/2072-666X/7/4/58/s1, Video S1: Example of locomotion of the MEMS microrobot system. Video S2: Heat conduction of the shape memory alloy-type rotary actuator. Video S3: Heat conduction of the new type rotary actuator comparing with old type. (Old rotary actuator.) Video S4: Heat conduction of the new type rotary actuator comparing with old type. (New rotary actuator.) Video S5: Movements of the rotor of the old type rotary actuator. Video S6: Movements of the rotor of the new type rotary actuator.
